# Advances in experimental psychopatholinguistics: What can we learn from simulation of disorder-like symptoms in human volunteers?

**DOI:** 10.2478/v10053-008-0137-6

**Published:** 2013-06-17

**Authors:** Stefan Heim

**Affiliations:** 1 Department of Psychiatry, Psychotherapy, and Psychosomatics, Medical School, RWTH Aachen University, Germany; 2 Research Centre Jülich, Institute of Neuroscience and Medicine (INM-1), Germany; 3 Section Clinical and Cognitive Neurosciences, Department of Neurology, Medical School, RWTH Aachen University, Germany; 4 JARA – Translational Brain Medicine, Jülich and Aachen, Germany

**Keywords:** aphasia, dyslexia, errors, simulation, language

## Abstract

For more than a century, work on patients with acquired or developmental language disorders has informed psycholinguistic models of normal linguistic processing in healthy persons. On the other hand, such models of healthy language processing have been used as blue-prints to gain further insights into the impairments of patients with language pathologies. Against the exemplary background of language production, the first part of this paper reflects this relationship and formulates a desideratum for naturalistic albeit controlled experimental settings. Two recent examples of behavioural and neurofunctional research are presented in which aphasia-like speech symptoms were elicited in healthy control subjects. In the second part, this idea to investigate disorder-like symptoms which are being experimentally induced for the course of the study is further pursued in the field of reading and dyslexia research. Here, it is argued, again on the basis of behavioural and neurofunctional data, that such an approach is advantageous in at least two respects:

1. It allows a much more stringent control of experimental factors and confounds than could be potentially achieved in a clinical setting.

2. It allows in-extenso piloting of experiments with healthy volunteers before actually recruiting selected (and sometimes rare) patients.

It will be concluded that the experimental simulation of disorder-like symptoms in easily accessible healthy volunteers may be a useful approach to understand novel aspects of a language disorder on the basis of a human neurocognitive model of this disorder.

## How can we model linguistic abilities?

Among human cognitive functions, our linguistic abilities are particularly fascinating. This is because, despite obvious differences between languages, basic principles such as the distinction between form (phonology) and content (semantics) can be identified, no matter which language a person speaks. As a scientific reflection of these regularities, linguistics has emerged as a research discipline, aiming to systematise the commonalities and particularities of languages. In its wake, psycholinguistics (i.e., the scientific approach to how linguistic principles are incorporated in the human cognitive architecture) became one of the psychological disciplines.

### What (speech) errors can tell us

Since the early observations of Broca (1861) and Wernicke (1874) that distinct lesions to the left hemisphere may cause distinct patterns of speech impairment, there has been an increasing interest in using speech error data in order to define and distinguish clinically relevant aphasia syndromes. Qualitative analysis of the patients’ speech samples (e.g., Huber, Poeck, Weniger, & Willmes, 1983) was combined with novel quantitative methods (Ash et al., 2010, 2012; Hussmann et al., 2012; Willmes, Poeck, Weniger, & Huber, 1983) to distinguish variants of aphasia or speech-related deficits based on standardised criteria. Moreover, the notion of systematic and reproducible error patterns gave rise to the idea that the cognitive mechanisms underlying normal language processing might at least partly be inferred from error patterns in patients (e.g., Geschwind, 1970; but see the early work by Jackson, 1879, for a critical discussion). Consequently, a number of models emerged that formalised aphasic symptoms while, at the same time, providing a potential reference frame for normal language processing (e.g., De Bleser, Cholewa, Stadie, & Tabatabaie, 2004; Kay, Lesser, & Coltheart, 1992; Morton, 1969; for a comprehensive review, cf. Coltheart, Rastle, Perry, Langdon, & Ziegler, 2001).

This logic was further adapted to also using speech error data from normal healthy speakers. For instance, the language production model of Gary Dell (Dell & O’Sheaghda, 1992; Dell et al., 1997) implemented this approach, thus being able to make assumptions about the interplay between distinct functional layers of nodes for semantic (meaning), lexical (word forms), and phonological (speech sound) processing. In this model, activation to any node(s) at any level(s) spreads between and within these levels. Connections between all nodes are bi-directional so that activation can flow to and fro. The most highly activated node finally gets selected. Speech errors may occur if either the nodes, or the connections between nodes, or both are impaired. Implementing the model architecture in a computer programme (Dell, Schwartz, Martin, Saffran, & Gagnon, 1997; Foygel & Dell, 2000; Saffran, Dell, & Schwartz, 2000; Schwartz, Dell, & Martin, 2006) now even allows allocating errors made by aphasic patients (e.g., during a picture naming task) to these different layers, thus providing a good estimate about the individual patient’s deficit (http://langprod.cogsci.illinois.edu/cgi-bin/webfit.cgi).

### Speed as a measure of unimpaired performance

Whereas Dell’s model is very convincing and helpful for describing speech errors, its scope is somewhat limited with respect to explaining normal, unimpaired language production. The features of normal language production are the core of an alternative approach by Pim Levelt (e.g., Levelt, 1989, 2001; Levelt, Roelofs, & Meyer, 1999), in which the relevant variable is not so much the type of speech error, but rather the speed at which a word is produced.^1^ The production speed is a relevant feature for this model because it directly relates to the model architecture. Different from Dell’s model, Levelt’s model has only uni-directional connections running in a one-way fashion from the idea to the spoken word. By virtue of this architecture, the speech latency reflects the speed at which entries at different processing levels get selected. Selectively manipulating the access to these features (e.g., by presentingauditory or visual distractors) therefore provides direct insight into the course of the production process and its duration at these different levels - which may be assessed in the range of milliseconds not only in the overt utterance but also in the electrophysiological scalp response using the lateralised readiness potential (LRP), a specific variant of event-related brain potentials (e.g., van Turennout, Hagoort, & Brown, 1997).

For experimental psycholinguistic work, the focus on speech latencies is advantageous because naming latencies can be obtained for every uttered word, be it correct or erroneous, whereas, for example, lexical selection errors tend to occur only at a rate of 1 out of 1,000 (Levelt et al., 1999).^2^ At the same time, different linguistic levels (e.g., semantic or phonological) can be addressed equally well as in Dell’s approach. Access to these levels can be manipulated, for example, by presenting distractor words which have certain relationship with the to-be-named picture (e.g., target: *cat*; semantically related: *dog*; phonologically related: *mat*). Since the early studies by Glaser and Düngelhoff (1984) and by Schriefers and colleagues (e.g., Meyer & Schriefers, 1991; Schriefers, 1993; Schriefers, Meyer, & Levelt, 1990), much work has been done to identify the influences of such distractors on lexical access. For instance, semantically related distractors tend to compete for lexical access with the target words, thus leading to interference. In contrast, phonologically related distractors are likely to facilitate the retrieval process by spilling over activation to shared phoneme nodes. These effects, however, may depend on, and change as a function of the context in which the utterance is produced (e.g., Hantsch, Jescheniak, & Mädebach, 2012; Jescheniak, Schriefers, & Hantsch, 2003).

Another advantage of using normal language production paradigms for modelling normal language production is that the investigations can be extended to phrases or even sentences, thus moving away from rather artificial single-word processing to more naturalistic settings. Doing so, one may investigate, for instance, whether the semantic or phonological information of a word in any particular slot of the syntactic sentence frame already becomes activated when the first word of the sentence is being uttered (e.g., Oppermann, Jescheniak, & Schriefers, 2010; Sass et al., 2010). This is an approach that partly supports the notion that during speech planning access to lexico-semantic concepts is incremental rather than owing to total in-advance preparation.

### Box-and-arrow models versus computational models

Performance of speakers, be they unimpaired or impaired, has been formalised in different ways in order to represent both types within the same framework. The classical approach used box-and-arrow models, representing a modular cognitive architecture (as, e.g., in Morton’s, 1969, logogen model). In this type of model, lesions were realised by selectively removing a box or an arrow, thus blocking processing along well defined pathways. This is effectively a binary manipulation with boxes and arrows being either intact (value: 1) or impaired (value: 0).

A more recent development was the parameterisation and computerisation of these kinds of models (e.g., Max Coltheart’s dual route cascaded model for reading: Coltheart et al., 2001; a parallel processing model by Sylviane Valdois’ group: Ans, Carbonnel, & Valdois, 1998; the online version of Gary Dell’s model [e.g., Foygel & Dell, 2000; Saffran et al., 2000]; or WEAVER++ by Ardi Roelofs [2000; passim] used for Levelt’s serial production model). These improved and parameterised (and thus non-binary) models can do two things. First, the parameters can be set such that the overall performance of the model resembles that of a given patient (e.g., Dell, Lawler, Harris, & Gordon, 2004; Dell, Martin, & Schwartz, 2007). Second, in theory, these parameters might also be manipulated not to match the performance of a real person, but rather to simulate qualitatively different conditions on the basis of quantitative parameter settings. Thus, symptoms or syndromes encountered in the real world could be predicted within a well-defined theoretical framework. Such predictions are valuable because they may refine our view of the world of empirical data, leading, for example, to the formulation of novel hypotheses about subtypes within a certain domain of functioning or disability.

### Speed versus accuracy, normal speakers versus patients

To summarise, what do these approaches imply for modelling the linguistic system in our minds? They demonstrate that data from patients are suitable for modelling the performance of patients, and likewise, that data from normal speakers may particularly inform models of normal speaking. Moreover, speech error data from patients and healthy participants allow some conclusion about the organisation of our linguistic abilities, whereas data from healthy controls mainly provide us with the (albeit very elaborate) notion of the normal state of affairs, rather than that of patients.

This situation is somewhat unfortunate, because healthy participants are much more easily available for psychological experiments. Moreover, they usually do not suffer from co-morbid disorders. Also, entire samples can be controlled for variables such as gender, age, intelligence, handedness, number of languages spoken, socio-economic background, etc. - all variables which likely affect performance and yet are much more difficult to control for in patient samples.

However, whereas sufficiently large samples of well-characterised patients may be hard to recruit^3^, the reverse problem arises for healthy volunteers. These although available in abundance, may not naturally be in a state to produce a sufficient number of symptoms.

Consequently, it would be desirable to investigate disorder-like symptoms in well-selected normal participants. Such investigations should produce effects which are comparable to those observed in real patients, both with respect to performance and (ideally) also to the underlying functional neuroanatomy.

The last part of this paper is dedicated to the presentation of two such simulation approaches, one from overt language production and one from reading. First behavioural studies evaluating the usefulness of these paradigms to elicit disorder-like symptoms in healthy, normal subjects are presented along with neuroimaging evidence that links the performance data to the brain.

## Novel simulation paradigms

### Trouble-indicating behaviour during continuous language production

Language production is not exactly error-prone. Rather, our performance in overt, continuous, every-day language is highly proficient, a fact that one might relate to built-in error-monitoring processors such as the self-monitoring loop assumed in Levelt’s (e.g., Levelt et al., 1999) model. Yet, not only the errors themselves, but also their correction may be reflected during speech production. Levelt (1983) distinguished five types of corrections or “repairs”: A-repairs (appropriateness corrections), C-repairs (covert), D-repairs (change of intention), E-repairs (error repairs), and R-repairs (non-classifiable). According to Levelt’s model described above, some of these errors could be taken as indication at which level of processing the original error occurred. A-repairs could reflect the selection of a wrong lexical entry in the course of speaking, for instance, the word *dog* if the sub-ordinate word *beagle* would be contextually more appropriate (e.g., in order to distinguish from a dachshund; “Do I want to say it this way?”, p. 51). In contrast, E-repairs might pertain to completed erroneousencoding which may be lexical or syntactic in nature. D-repairs indicate that the speaker realises in the course of speaking that another arrangement of the utterance would be more appropriate (“Do I want to say this now?”, p. 51). In addition to this classification of repairs, Schlenck, Huber, and Willmes (1987) provided a wider taxonomy of what they termed “trouble indicating behaviour”, a concept subsuming not only repairs but also “prepairs” involving hesitations, word retrieval difficulties, and circumlocutions. Levelt’s (1983) idea of an internal monitor which contributes to the repairs was expanded and tested in a computer simulation by Hartsuiker and Kolk (2001), with findings corroborating this idea (see Nozari, Dell, & Schwartz, 2011, for a novel model of speech monitoring mechanisms that are based within the language production system and do not rely on the comprehension system; for an earlier review of different notions of monitors, cf. Postma, 2000).

Recently, a couple of paradigms were presented in order to elicit in healthy speakers such trouble-indicating behaviour in sufficient amounts to mimic that of patients. For instance, Hodgson and Lambon Ralph (2008) introduced a speeded picture naming task intended to elicit semantic errors. Similarly, speeded responses were also required in the study by Moses, Nickels, and Sheard (2004), which led to perseveration errors during overt reading and naming. The interesting aspect of these two studies was that (differently than in, e.g., the picture-word interference paradigm) these errors had no direct trigger in the sense that no other stimulus was presented that would have interfered with the retrieval of the correct lexical-semantic or phonological information. This property makes the performance more comparable to that during natural speaking, since errors, repairs, or prepairs occur on the fly.

A further step towards a naturalistic situation would be if the utterance format was not words but sentences within one coherent text. This approach was pursued by Elisabeth Meffert and colleagues (2011), who in their Experiment 2 asked their healthy volunteers to describe black-and-white line drawings of complex real-life scenarios (e.g., at the train station, in a car accident on a street, in the hospital, etc.). On each picture, many sub-scenarios could be detected. The participants had a total of 3 min to describe aloud what they could see on the picture, leaving it open to them with which part of the scene to start. However, they were instructed not to use a handful of keywords written on the upper and lower rim of the picture. These keywords were derived from Experiment 1, in which the number and type of propositions contained in each picture had been identified. The keywords thus referred to topics that were likely to be mentioned while describing the picture, and probably with exactly this word. For instance, in a picture showing a scene at a circus, the forbidden words were *tiger - circus - elephant - clown - saxophone*, all of which were present on the picture.

The spontaneous speech of all participants was recorded and analysed in two ways. First and most importantly, different types of trouble-indicating behaviour (Schlenck et al., 1987) were assessed and compared between the two experiments. Second, a formalised analysis tool (ASPA; Huber, Grande, & Springer, 2005; Hussmann et al., 2006) was applied to assess basic parameters of spontaneous speech (e.g., mean length of utterances; type-token ratio; percentage of items of open word class). As expected, Meffert et al. (2011) could demonstrate that the amount of trouble-indicating behaviour increased significantly in Experiment 2 when subjects had to avoid the target keywords. A further analysis corroborated that the subjects in Experiment 2 did not produce more unspecific trouble-indicating behaviour (like interjections “er”, “um”, etc.), what might reflect a higher overall difficulty of the task in which the forbidden keywords had to be checked repeatedly. Rather, it was really specific trouble-indicating behaviour associated with difficulties of lexical retrieval that was increased in Experiment 2. Interestingly, the most frequent type of trouble-indicating behaviour was prepairs, which might not figure in classical single-word naming experiments. These prepairs may be taken to indicate the successful correction of a lexical selection error at the concept or lemma level before the actual lexeme was retrieved. The second-most produced category were C-repairs which, according to Levelt (1983), are difficult to interpret because they are, by definition, covert. Still, these indicate that the repair was completed at least before the initiation of the articulation, that is, somewhere between the concept level and the lexeme level. Importantly, most of these C-repairs occurred in relation to forbidden words, demonstrating that these were indeed the source of the repair. Moreover, the overall greater proportion of C-errors than A-errors, D-errors, or E-errors indicates that the internal monitoring mechanism was unaffected by the experimental manipulation.

Together, these findings demonstrated that the use of forbidden keywords is a promising approach for challenging overt language production which induces specific trouble-indicating behaviour like in fluent aphasic patients. Methodologically, the study provided a total of nine such stimulus pictures, which were highly comparable with respect to linguistic parameters and which may thus even be used for repeated testing of subjects with parallel test forms.

To test comparability between real aphasic patients and subjects undergoing this simulation paradigm, a complex schema for analysis of the entire continuous speech sample on a second-by-second basis was developed and administered both to an aphasic patient (Tillmanns et al., 2011) and to healthy speakers (Grande et al., 2012) while performing the picture description task in a functional magnetic resonance imaging (fMRI) scanner. This second-by-second approach could be used to classify each moment during speaking as impaired or unimpaired, with further sub-classifications along the dimensions of monitoring (present/absent), success of the outcome of a retrieval phase (successful/not successful), and the (pre-)lexical category of the symptom. The study by Grande et al. revealed that indeed Meffert’s simulation paradigm activates the well-known left-lateralised fronto-parieto-temporal speech network in the brain. Moreover, the second-by-second event classification schema could be used to dissociate phases of conceptual planning of the upcoming sentence (recruiting mental imagery areas of the visual cortex) from those of lexical retrieval (left middle temporal gyrus) or syntactic encoding (Area 44 in Broca’s region).

Application of this paradigm to a fluent aphasic patient (Tillmanns et al., 2011) further revealed how the language system is affected, and gets re-organised, relatively to the situation in healthy controls. The patient, a 53 years old woman, had a left posterior-temporal lesion which caused mild fluent aphasia with predominant word finding difficulties. Comparing her unimpaired language production against a resting baseline revealed a seemingly normal left-hemispheric activation pattern with additional activation in right-hemispheric homologue areas. However, lexical search, which relies on the posterior middle temporal cortex in which her lesion was located (e.g., Whitney, Kirk, O’Sullivan, Lambon Ralph, & Jefferies, 2011), was characterised by relatively increased involvement of left and right supramarginal and angular gyri. Whereas the involvement of the left inferior parietal cortex is in line with the literature, the strong activation in the right inferior parietal lobule stands out against findings from healthy controls (Vigneau et al., 2011), stressing the importance of right-homologue areas for quasi-successful compensation of word finding difficulties.

To summarise, Meffert’s paradigm seems to be a promising approach to elicit aphasia-like speech errors in healthy volunteers with well-defined demographic parameters in continuous overt sentence production which informs the analysis of the functional neuroanatomy of aphasia.

### Cognitive fingerprints of developmental dyslexia

The focus of the first paradigm was to elicit linguistic behaviour in healthy controls that would resemble that of real patients. The focus of the second paradigm, which will be introduced in this chapter, goes even further in that the behaviour of subgroups of patients is targeted. The starting point for this endeavour was the finding that reading problems in developmental dyslexia may evolve from very distinct underlying cognitive profiles. As Ramus (2003, 2004) pointed out, not all dyslexic readers profit from the same kind of intervention. Moreover, they may present different patterns of cognitive problems, which could be visual, phonological, auditory, etc. These reports led to large-scale investigations of cognitive subtypes of developmental dyslexia (Heim et al., 2008; Heim, Grande, Meffert, et al., 2010; Heim, Grande, Pape-Neumann, et al., 2010) which indeed revealed two major processing pathways relevant for reading: one phonological and one visual. Studies in the cognitive-behavioural domain (Aguilar Isaías, 2006; Bosse, Tainturier, & Valdois, 2007; Heim et al., 2008; Ramus et al., 2003; Reid, Szczerbinski, Iskierka-Kasperek, & Hansen, 2007; Valdois et al., 2003) as well as neuroimaging findings (Danelli et al., 2012; Heim, Grande, Meffert, et al., 2010; Heim, Grande, Pape-Neumann, et al., 2010; Peyrin et al., 2012; Savill & Thierry, 2012) provided evidence that these pathways can be selectively impaired, while impairment may also affect both.

Another experience from these studies was that recruiting dyslexic children may be a tedious endeavour because of their very limited spare time, which is even more limited if they receive some kind of remediation training. Likewise, a substantial proportion of children refused fMRI scanning or quit during the measurement. On the basis of these experiences, the idea emerged that piloting of new experiments might be done without the involvement of real dyslexics. If phonological versus visual processing problems could be introduced in experiments with healthy readers, such findings could be generated in extenso before actually recruiting real dyslexics.

The simulation paradigm that Tholen, Weidner, Grande, Amunts, and Heim (2011) came up with considered both empirical findings and self-reports from dyslexics, which repeatedly stated that their visual percept of letters was not stationary but moving or turning. Based on these reports, written words and pseudowords were manipulated such that their letters might be dancing in a vertical movement of varying amplitude and phase. This manipulation interfered with the entire shape or “visual word form” of the stimulus, and likewise impaired visual scanning from one letter to the next. For a phonology-based manipulation, stimuli were presented in an unfamiliar though clearly identifiable font, which made the conversion of graphemes to phonemes more difficult. A control analysis indeed revealed that processing in this simulation depended on the phonological awareness of the participants (which was tested with a standardised German battery, viz., BAKO; Stock, Marx, & Schneider, 2003).

The participants’ task was a visual lexical decision, pressing the left or right response button to indicate whether the stimulus was a word or a pseudoword. Like in real dyslexia, both reading speed and reading accuracy were impaired independently in both experimental conditions. Moreover, in the phonology-near condition, pseudoword processing was affected in particular, mirroring the widely-reported fact that (phonological) dyslexics have these particular difficulties (e.g., Lallier, Donnadieu, Berger, & Valdois, 2010; but see Lachmann, Berti, Kujala, & Schröger, 2005, for controversial discussion). Thus, a paradigm was created that could experimentally manipulate reading performance along two routes - something that could previously only be simulated in computer models of reading (e.g., Coltheart et al.’s, 2001, dual route cascaded model) but not in real experimental data.

Setting up such a paradigm was only the first step, which should be accompanied also by neurofunctional data. If the paradigm was a good simulation of (different types of) dyslexic reading, this should also be reflected in the brain regions involved in each of the conditions, which would ideally match those found in real dyslexic subjects (Heim, Grande, Pape-Neumann, et al., 2010). Consequently, an fMRI study was conducted with a novel sample of healthy readers (Heim et al., 2013; see also Heim & Grande, 2012). While replicating the previously obtained behavioural findings of Tholen et al. (2011), the study also revealed involvement of the expected brain areas, that is, left and right area V5 of the visual-magnocellular pathway for the visual condition, and a left fronto-parietal network for the phonology-near condition. Interestingly, there was also right-hemispheric involvement in the phonological condition. This latter result inspired the novel hypothesis that right fronto-parietal homologue areas might be involved in compensation processes in dyslexia - an assumption that is in line with earlier findings by Heim, Grande, Meffert, et al. (2010) who observed stable recruitment of these areas in dyslexic but not in normal readers. To conclude, Tholen’s paradigm was useful to elicit dyslexia-like reading behaviour in healthy volunteers with well-defined demographic parameters along two cognitively and neurofunctionally distinct processing pathways and provided novel hypotheses about the role of the right hemisphere in reading and dyslexia.

### Virtual lesion models

Recent technical advances now provide the possibility to simulate deficits in healthy volunteers by temporary virtual lesions to their brains. These can be administered, for example, by transcranial magnetic stimulation (TMS) or by transcranial direct current stimulation (tDCS). In short, depending on the actual parameters, these techniques directly or indirectly affect the excitability of neural populations in a relatively well defined patch of cortex, thus impairing the functionality of this part of the brain in a given cognitive context. There is an abundance of well conducted studies using these techniques which provide meaningful and reliable data, which cannot be reviewed in due depth here. One example relevant to the topic of this paper is the tDCS study by Pisoni et al. (2012) who investigated the role of the left inferior frontal gyrus for the semantic interference effect discussed above. Complementary data stressing the role of other cortex areas intimately related to the left inferior frontal gyrus were provided in the TMS studies by Thiel et al. (2005) and by Whitney et al. (2011). Thiel et al. investigated the interplay of the left inferior frontal cortex and its right homologue in healthy volunteers and in tumour patients. Whitney and colleagues were able to show the complementary role of the left posterior middle temporal gyrus, which is functionally linked to the left inferior frontal cortex during semantic selection (cf. the match to the data obtained by Tillmanns et al., 2011). Likewise, in the field of reading and dyslexia, TMS has proven useful to understand the role of the left occipitotemporal cortex in visual word recognition (e.g., Devlin & Watkins, 2007; Duncan, Pattamadilok, & Devlin, 2010).

There are two reasons why this virtual lesion approach is not discussed in depth in the present paper. The one is that TMS or tDCS are not as easily available to the *cognitive* (neuro-)scientist. (Admittedly, fMRI technology may be likewise unavailable to a cognitive neuro-scientist. The reason why fMRI data have been discussed in this paper was foremost to provide additional evidence independent of pure behavioural data which demonstrates that the simulations provide reasonable results.) The other is that the virtual lesion approach has the same strengths and drawbacks as research in real lesion patients: Inferences about cognitive processes are made on the basis of anatomical facts or manipulations, not on the basis of cognitive manipulations. Ideally, a neuroscientist would use different approaches (like cognitive simulations, virtual lesions, and neuroimaging techniques) in combination in order to obtain comprehensive data about successful and impaired language processing, which, in turn, may be useful for the development of effective remedies for patients with language disturbances.

## Conclusion

The present paper has discussed psychopatholinguistic studies of impaired and unimpaired behaviour, the bottom-line being a trade-off between the availability of well-characterised patients on the one hand, and on the other hand, the performance of healthy volunteers that does not exactly reflect the symptoms of patients. As a possible solution, the use of simulation paradigms to elicit disorder-like symptoms in healthy volunteers for the course of the experiment has been suggested. The paper has given two examples of useful simulation paradigms from the domains of language production/aphasia and reading/dyslexia. It should be noted that the logic and mechanisms in these two examples were slightly different. In the first example, processing was impaired by presenting an extra obstacle (i.e., not being allowed to produce some words that likely come to mind) - a phenomenon also encountered in aphasic patients when they note that they retrieved the wrong word and seek to come up with the appropriate one. Thus, the problem here has a top-down processing component for the subjects. In the second example, the correct target was unknown to the subjects, and the difficulties here were of a bottom-up nature (distortion of the visual signal or impoverished knowledge about the sound value of a letter shape) than in the first example. Together, this simulation approach reflected in the two examples should be regarded as a complement, rather than a competitor, to work with real patients. Indeed, it would seem reasonable to pilot novel ideas with healthy volunteers in such simulation paradigms in order to get some preliminary experience with the data before recruiting real patients to undergo procedures which may be stressful and maybe also emotionally painful for them in cases of failure. Such integration of multiple sources of information from intact and impaired behaviour of patients and healthy volunteers would certainly reflect an advance in experimental psychopatholinguistics.

## Footnotes

1 Note that Levelt et al. (1999) provide a very balanced view of the advantages of speech latencies and speech errors, and acknowledge the previous work, for example, by Shattuck-Hufnagel (1979, 1983, 1985). Levelt et al. agree that any model of speech production should be able to account for speech errors. In Section 10 of their paper, they therefore explain that the underlying computer model WEAVER++ does not make any errors in its basic version. Still, there are several sources for errors. For instance, the lack of feedback connections from the lexeme to the lemma level may lead to delayed corrections if a phoneme exchange results in a real, semantically related word (e.g., *rat* instead of *cat*). Another possibility is that, in rare cases, two target lemmas get selected (e.g., *close* and *near*), which may then blend into the existing word *clear*.

2 There are techniques to increase the normal proportion of speech errors, for example, the SLIP technique (Baars, Motley, & MacKay, 1975). Here, word pairs are presented in which the first word always starts with Phoneme 1 (e.g., /*b*/) and the second word with Phoneme 2 (e.g., /*d*/). After a series of such pairs (*book-deer*, *bear-drug*, *bee-dung*; etc.), a word or non-word pair with reversed initial phonemes (e.g., *darn-boor*) is presented. Upon such stimuli, subjects tend to remain in the habit of producing the first item with /*b*/ and the second with /*d*/, resulting in an error (in this case the lexicalisation error *barn-door*). Note that overt picture naming has several advantages over other elicitation procedures such as, for instance, word naming (cf. Indefrey & Levelt, 2000): 1. The lexical concept can be accessed without interference from orthographic processing. This fact is in particular relevant in languages with low orthographic transparency (such as English) where homograph words may have different readings (e.g., *read*, *wind*, *lead*). Likewise, the examination of speech performance of stroke patients would be confounded by their remaining orthographic decoding abilities. 2. In contrast to generation procedures such as verbal fluency or word association, the picture naming task does not have to make assumptions about associative strengths or the executive components supposed to underlie the word fluency procedure.

3 One potential solution to this dilemma is the use of case series (for a recent instructive review, see Schwartz & Dell, 2010) instead of single or multiple case studies (e.g., Etcheverry et al., 2012). The case series approach stands in opposition to the classical neuropsychological approach. Whereas the latter basically regards the (often unavoidable) heterogeneity in a group of patients as a source of noise (i.e., as a nuisance variable), the case series approach effectively takes variability as a source of information. Within this controlled setting, associations between different linguistic or cognitive domains can be assessed, for example, via regression techniques, provided the sample size is sufficient (*n* > 10). As Schwartz and Dell (2010) pointed out, the case series approach may be advantageous because it has the potential to reconcile on the basis of quantitative analysis positions that qualitatively seem contradictory. For example, the fact that some stroke patients produce predominantly semantic errors while others mostly make non-word errors might be taken to suggest a quantitative distinction between subgroups of patients. However, as Dell et al. (1997) demonstrated, considering the overall severity of symptoms as a random variable revealed that both types of errors were related to, and could thus be regarded as differential expressions of this overall severity - and that the patients were only part of the same continuum.
